# Perioperative multidisciplinary rescue of a patient with Erdheim - Chester disease and multi-system involvement: a case report

**DOI:** 10.3389/fmed.2026.1725570

**Published:** 2026-01-29

**Authors:** Zhongyu Wang, Shuang Yin, Lichang Wang, Bucheng Liao

**Affiliations:** 1 Department of Anesthesiology, Peking University Shenzhen Hospital, Shenzhen, Guangdong, China; 2 Department of Anesthesiology, Shenzhen Hospital, Southern Medical University, Shenzhen, Guangdong, China

**Keywords:** case report, Erdheim - Chester disease, multidisciplinary, peri-operative management, rare disease

## Abstract

Erdheim - Chester disease (ECD) is a rare, slowly progressive non-Langerhans cell histiocytosis that affects bones and multiple viscera. In 2016 the World Health Organization classified ECD as a distinct entity within the histiocytic neoplasm family. Fewer than 1500 cases have been documented in the international literature and the ECD Global Alliance registry. We report a 49-years-old man with biopsy-proven Erdheim - Chester disease (ECD) who developed acute suppurative appendicitis. After systematic evaluation he underwent laparoscopic appendectomy under general anesthesia and, because of multi-organ ECD involvement, was transferred to the intensive care unit (ICU). A multidisciplinary regimen of lung-protective ventilation, continuous renal-replacement therapy, broad-spectrum antibiotics, granulocyte colony-stimulating factor and glucocorticoids was instituted, leading to stable respiratory and renal function; he was discharged to the hematology ward on post-operative day 10. ECD patients are prone to peri-operative deterioration due to pulmonary infiltration, refractory hypoxemia, acute kidney injury and immune paralysis; successful outcome depends on coordinated multidisciplinary care, precise hemodynamic and volume management, early continuous renal replacement therapy (CRRT) and immunomodulation.

## 1 Introduction

Erdheim- Chester disease (ECD) was first described by Erdheim and Chester in 1930 (1). Histologically, it is characterized by sheets of lipid-laden, foamy histiocytes that infiltrate bone, cardiovascular structures, lungs, kidneys, brain, hypothalamo - pituitary axis, retroperitoneum, orbit and skin, inciting a fibrotic reaction. Approximately 50% of patients harbor the BRAF V600E mutation; BRAF encodes a RAS/MAP - kinase signaling protein that regulates proliferation, differentiation and apoptosis. The mutation is somatic, confined to lesional histiocytes or their immature precursors, and results in constitutive BRAF activity, uncontrolled clonal expansion and organ accumulation (2). Additional MAPK - pathway variants (MAP2K1, NRAS, KRAS, ARAF) have also been implicated (3). Consequently, ECD is now regarded as a clonal, inflammatory myeloid neoplasm driven by MAPK activation rather than a purely inflammatory disorder (4). Here we report the peri-operative management of an ECD patient with multi-organ involvement who underwent appendectomy and review the clinical features and therapeutic experience of previously published cases.

## 2 Case presentation

**FIGURE 1:**
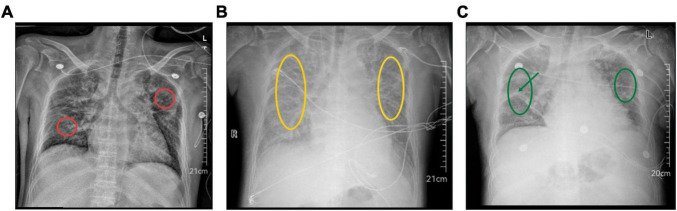
Perioperative chest radiographs. **(A)** Pre - operative film: bilateral interstitial pneumonia with reticular changes (red circle). **(B)** Post - operative day 3: bilateral interstitial pneumonia complicated by pulmonary edema; both lung fields show decreased translucency and diffuse opacities (yellow circles). The right-lung lesion has progressed compared with the pre-operative film, whereas the left-sided changes remain essentially unchanged. At this point the patient had received 500 mL of intravenous crystalloid plus 1 L oral intake, had become anuric. **(C)** Post - operative day 17: bilateral interstitial pneumonia with persistent exudation; both lung fields remain hypolucent, exudative shadows (green circles) and inter-lobar septal lines (green arrow) are visible. The right-lung lesion has improved compared with the day-3 film, while the left-sided changes are still essentially unchanged. At this point the patient had been transferred to the hematology ward after completion of continuous renal-replacement therapy.

A 49 - years - old man was admitted with a 3 - day history of right-lower-quadrant pain and fever. Nine years earlier he had been found to have chronic neutropenia that had failed to respond to 1 year of cyclosporine and glucocorticoid therapy; since then he had remained off therapy with persistently low white-cell counts. One year before the current admission he had developed polydipsia - polyuria and had been diagnosed with central diabetes insipidus, which was well controlled with desmopressin. Hypertension had been recognized at the same time and was adequately controlled with bisoprolol 2.5 mg once daily. Type 2 diabetes mellitus had been diagnosed 3 months earlier; he had taken metformin and insulin for 2 months but had then discontinued both medications. One month before admission whole-body Positron Emission Tomography - Computed Tomography (PET - CT) had shown symmetrically intense osseous uptake; histopathology of the femoral lesion revealed diffuse infiltration of foamy histiocytes between mature trabeculae, with focal bone destruction and marked stromal fibrosis. Immunohistochemistry showed diffuse positivity for CD68 and CD163, and BRAF-V600E was positive. Next-generation sequencing of the same formalin-fixed tissue verified the BRAF-V600E mutation, confirming the diagnosis of ECD involving bone, central nervous system (CNS), heart, kidneys and lungs. He had improved on conservative therapy and had been discharged. On presentation this time his white-cell count was 0.11 × 10^9^/L (neutrophils: 0.01 × 10^9^/L), hemoglobin 84 g/L, N - terminal pro-brain natriuretic peptide (NT-pro-BNP) 865 pg/mL, Procalcitonin (PCT) 6.41 ng/mL and interleukin - 6 (IL-6) 91.7 pg/mL. Chest radiography showed interstitial lung changes consistent with ECD - related pulmonary infiltration (Figure 1A). Contrast-enhanced abdominal Computed Tomography (CT) revealed an enlarged appendix containing a faecolith and surrounded by fat stranding, circumferential thickening of the ascending colon, and bilateral hydronephrosis with mural thickening of the renal pelvis and proximal ureters, all suggestive of active inflammation. Admission diagnoses were (1) acute suppurative appendicitis, (2) localized peritonitis, (3) ECD with skeletal, central-nervous, cardiovascular, renal and pulmonary involvement, (4) agranulocytosis, (5) central diabetes insipidus, (6) bilateral interstitial pneumonia, (7) hypertension and (8) type 2 diabetes mellitus.

The patient underwent emergency laparoscopic appendectomy under general anesthesia. On arrival in theater he was drowsy but oriented, 177 cm tall and 68 kg. Baseline peripheral capillary oxygen saturation (SpO2) was 92%–95% on 5 L/min oxygen via facemask; blood pressure 122/79 mmHg and heart rate 99 beats/min. A right-radial arterial line was inserted under local anesthesia. Anesthesia was induced with ciprofol 25 mg, rocuronium 50 mg, sufentanil 20 mcg and dexamethasone 5 mg and maintained with 2% sevoflurane plus remifentanil 0.1 mcg/kg/min. Intra-operatively, a lung-protective strategy was used: tidal volume 7 mL/kg ideal body weight, and positive end - expiratory pressure (PEEP) 5 cm H_2_O; FiO_2_ was progressively increased to 100% to keep SpO_2_ 95%–98%. Noradrenaline infusion was required to maintain mean arterial pressure 70–80 mmHg. Methylprednisolone 40 mg was given intravenously. Serial arterial blood-gas analyses (Table 1) revealed combined respiratory and metabolic acidosis, which was corrected by infusion of 5% sodium bicarbonate and by increasing the respiratory rate to augment minute ventilation. Arterial blood gas obtained shortly before leaving the operating room showed improved acid - base status. Surgery lasted 65 min; blood loss was 5 mL, urine output 600 mL and crystalloid infusion 1100 mL. Histopathological examination of the resected appendix revealed mucosal ulceration, vascular congestion, and infiltration of neutrophils, lymphocytes and plasma cells within the wall, consistent with acute suppurative appendicitis. The trachea was kept intubated and the patient was transferred to the intensive care unit (ICU) for further management.

**TABLE 1:** Intra-operative arterial blood-gas data.

Time point	FiO2 (%)	pH	PaO2 (mmHg)	PaCO2 (mmHg)	BE (mmol/L)	HCO3^–^ (mmol/L)	Lactate (mmol/L)
25 min after incision	100	7.144	103	49.9	−11.5	15.4	0.6
56 min after incision	100	7.192	111	53.0	−7.9	18.1	0.6
10 min before leaving OR	100	7.220	116	49.3	−7.8	20.3	0.6

Data are single-point measurements. FiO2, fraction of inspired oxygen; PaO2, arterial partial pressure of oxygen; PaCO2, arterial partial pressure of carbon dioxide; BE, base excess; HCO3^–^, bicarbonate; OR, operating room.

In the ICU he remained sedated and was ventilated in synchronized intermittent mandatory ventilation (SIMV) mode. PCT fell but IL - 6 continued to rise; empirical meropenem 2 g 8-hourly was started. Diabetes insipidus produced 5390 mL urine in 24 h with hypernatremia and hypokalemia, corrected with 0.9% saline plus potassium chloride and subcutaneous desmopressin while maintaining a mild negative fluid balance. To correct agranulocytosis, granulocyte colony-stimulating factor (G - CSF) was administered at 300 mcg daily. The endotracheal tube was removed on post-operative day 1 and the patient was transferred to the gastrointestinal surgical ward on day 2.

On the day of transfer to the gastrointestinal surgery ward, the patient experienced sudden volume overload (500 mL intravenous crystalloid plus 1 L oral fluid intake) but remained anuric. Despite administration of oral furosemide 20 mg and intravenous furosemide 10 mg, urine output was only 20 mL. The patient presented with dyspnea and progressive hypoxemia (SpO2: 84%–90%) and was directly returned to the ICU. Chest X - ray revealed bilateral interstitial pneumonia with progression of right-sided pulmonary lesions (Figure 1B). The hypoxemia was attributed to ECD involving the lungs, complicated by acute kidney injury (AKI), volume overload, and bilateral pleural effusions. Non-invasive ventilation and high - flow nasal cannula oxygen therapy were alternately administered. Following continuous renal replacement therapy (CRRT), the patient’s respiratory status stabilized, with an oxygenation index of 405 mmHg, and oxygen therapy was downgraded to nasal cannula.

Representative chest radiographs obtained pre-operatively and on post-operative days 3 and 17 are shown in Figure 1, demonstrating bilateral interstitial pneumonia with gradual improvement in the right lung.

The patient presented with anuric onset and AKI. Renal ultrasonography showed no mechanical obstruction, whereas abdominal CT revealed bilateral renal enlargement, thickening of the renal pelvis and ureteral walls, and hydronephrosis. Serum creatinine and NT - pro - BNP levels were significantly elevated. The renal dysfunction was possibly related to ECD involvement of the urinary system, postoperative stress, and infection. After CRRT and corticosteroid therapy to reduce fluid overload, urine output increased, and cardiac injury markers showed a downward trend. CRRT was discontinued after 2 days.

Peritoneal drainage fluid culture grew *Enterococcus faecium*. Although inflammatory markers remained elevated, they showed a declining trend. Given the patient’s neutropenia and immunocompromised status, antimicrobial therapy was broadened to cover *Enterococcus faecium*, fungi, and anaerobes. The patient received intravenous tigecycline 50 mg every 12 h and caspofungin acetate 50 mg once daily.

Notably, the patient remained persistently neutropenic. Despite G - CSF administration, neutrophil count increased only modestly. Dexamethasone 5 mg was added to suppress inflammation and promote granulocyte recovery, after which leukocyte counts gradually improved. Given the improved infection status, corticosteroid therapy was escalated to intravenous methylprednisolone 40 mg daily, in combination with recombinant G-CSF 300 mcg daily for leukocyte stimulation.

By post-operative day 10 the patient was afebrile, hemodynamically stable and free of dyspnea, expectorating small amounts of yellow-white sputum while breathing 4 L/min nasal oxygen (SpO2: 97%). Inflammatory indices were acceptable and chest auscultation demonstrated only faint bibasal crackles. He was transferred to the general hematology ward for ongoing neutrophil recovery and tailored anti-infective therapy. Upon transfer, the patient reported satisfactory comfort and denied any further symptoms. The peri - operative laboratory trends are summarized in Table 2, illustrating the gradual improvement in infection markers, cardiac strain, and persistent agranulocytosis. The chronological sequence of key peri-operative events is provided in Table 3.

**TABLE 2:** Perioperative laboratory results.

Parameter	Pre-op	POD 1	POD 3	POD 6
PCT (ng/mL)	6.41	5.03	3.78	0.66
IL-6 (pg/mL)	2756	385	443	14.3
NT-pro-BNP (pg/mL)	865	1009	16883	5130
Neutrophils (×10^9^/L)	0.01	0.01	0.02	0.02

PCT and IL-6 track infection control; NT-proBNP indicates cardiac strain; neutrophil count reflects perioperative agranulocytosis. PCT, Procalcitonin; IL-6, Interleukin-6; NT-pro-BNP, N-terminal pro-brain natriuretic peptide.

**TABLE 3:** Peri - operative time course of the Erdheim - Chester disease patient undergoing emergency appendectomy.

Post - operative day	Time	Key events
POD 0	18:00	Emergency laparoscopic appendectomy completed; the patient remained intubated and was transferred to the ICU.
POD 0	19:40	Meropenem and G-CSF were initiated.
POD 1	08:30	Endotracheal tube removed; spontaneous breathing resumed; desmopressin administered for central diabetes insipidus.
POD 2	10:30	Transferred to gastrointestinal surgery ward; 500 mL crystalloid infused and 1 L oral water allowed.
POD 2	23:00	Anuric acute kidney injury with fluid overload unresponsive to furosemide; readmitted to ICU; chest film showed progression of bilateral interstitial pneumonia.
POD 3	08:50	Intravenous glucocorticoids (dexamethasone and methylprednisolone) added.
POD 3	13:00	CRRT initiated.
POD 3	18:00	Peritoneal-fluid culture yielded *Enterococcus faecium*; antibiotics switched to tigecycline and caspofungin.
POD 5	12:00	Urine output increased; CRRT discontinued.
POD 10	13:30	The patient hemodynamically stable and was transferred to hematology ward for further leukocyte recovery and infection management.

POD, post-operative day; ICU, intensive care unit; G-CSF, granulocyte colony-stimulating factor; CRRT, continuous renal-replacement therapy.

## 3 Discussion

Erdheim - Chester disease may involve virtually any organ and carries a poor prognosis. Onset is typically between the fourth and sixth decades, although severity ranges from asymptomatic indolent disease to life - threatening multi-organ failure. Manifestations are site - dependent and non-specific; bones, CNS, orbit, hypothalamo-pituitary axis, lungs, heart, kidneys and retroperitoneum are most frequently affected (5). Common clinical manifestations of ECD by organ system are outlined in Table 4, highlighting the multisystem nature of the disease. Our 49 - years - old patient exemplified this systemic tropism, presenting with interstitial lung disease, myocardial involvement (NT-proBNP 865 pg/mL), hydronephrosis due to ureteric wall thickening and central diabetes insipidus from hypothalamo - pituitary axis injury.

**TABLE 4:** Erdheim - Chester disease (ECD) organ-system involvement – clinical manifestations.

System/organ	Common symptoms/complications
Bones	Limb pain, osteosclerosis
CNS	Raised ICP, headache, seizures, cognitive/motor/sensory deficits
Hypothalamic–pituitary axis	Endocrine dysfunction: ADH secretion deficit causing central diabetes insipidus
Respiratory	Interstitial lung disease, fibrosis, dyspnea, respiratory failure
Cardiovascular	Pericardial thickening/effusion, myocardial damage, valvular dysfunction, arrhythmia, heart failure
Vascular	Perivascular infiltration with stenosis and organ ischemia
Renal	Renal hypertension, urinary obstruction, renal failure
Other	Exophthalmos, skin lesions, systemic symptoms (fever, weight loss, night sweats)

ECD, Erdheim - Chester disease; CNS, central nervous system; ICP, intracranial pressure; ADH, antidiuretic hormone.

Diagnosis is established by integrating clinical manifestations with characteristic imaging findings - symmetric, diaphyseal-metaphyseal osteosclerosis of long bones exhibiting intense 99mTc uptake on bone scintigraphy and histopathological confirmation of lipid-laden, non-Langerhans histiocytes that are CD68^+^/CD1a^–^/S100^–^ (6). Therapeutic armamentarium includes interferon - α, glucocorticoids, cyclosporine, conventional chemotherapy, the BRAF inhibitor vemurafenib, surgical resection and radiotherapy (7); the advent of targeted therapy has reduced 3-years mortality from 60% to 20% (7, 8).

It should be noted that Franconieri et al. (9) previously reported two patients with intra-abdominal Erdheim - Chester disease (ECD) who presented with imaging findings mimicking “pseudotumoral appendicitis” and mesenteric panniculitis; both showed marked radiologic thickening of the appendix and mesenteric fat layers and achieved complete or partial remission after interleukin - 1 (IL - 1) blockade with anakinra, without the need for surgical resection. Our patient differed in three respects: (1) CT confirmed acute appendicitis with an appendicolith; (2) the peripheral neutrophil count was almost undetectable (0.01 × 10^9^/L); and (3) overt peritonitis and the high risk of fecal perforation under agranulocytosis mandated immediate surgery. These distinctions underscore that, although IL - 1 inhibition may suffice for purely inflammatory ECD pseudotumors, emergent appendectomy remains mandatory when bacterial infection and luminal obstruction coexist. Moreover, chronic neutropenia can attenuate classic inflammatory signs and delay diagnosis. In the present case, the combination of a CT - documented faecolith, peri-appendiceal exudate, and rising inflammatory markers prompted immediate laparoscopic appendectomy; postoperative histopathology confirmed acute suppurative appendicitis. Of note, diffuse infiltration of foamy histiocytes within the appendiceal tissue was not observed.

Successful peri-operative care hinges on systematically identifying and quantifying the function of every potentially affected organ before surgery. Because central nervous, respiratory and cardiovascular involvements are the leading causes of death, they warrant particularly scrupulous evaluation. Clinical reports on anesthetic management of this disease remain extremely scarce. Hariharan (10) described a single case of open reduction and internal fixation of an ankle fracture performed under continuous epidural anesthesia, emphasizing that the multi-system burden of ECD poses a formidable challenge to anesthesiologists and demands meticulous attention to every detail. Recommended precautions include pre-operative pulmonary optimization (physiotherapy, bronchodilators and readiness for post-operative ventilation), assessment of renal dysfunction with appropriate anesthetic-drug adjustments and provision for intra-operative dialysis, continuous monitoring for conduction defects, cardiac tamponade or heart failure, strict fluid balance and early recognition and treatment of diabetes insipidus, protection of pressure points and bony prominences, screening for spinal deformity before neuraxial block, ophthalmological evaluation to document optic-nerve compression and avoidance of increases in intra-ocular pressure, and detection and replacement of endocrine deficits arising from hypothalamo-pituitary injury-most notably peri-operative hydrocortisone for possible adrenal insufficiency and desmopressin for central DI - while remaining vigilant for side-effects of chronic medications.

We should also not neglect intra - operative anesthetic management. This patient presented with ECD-related multi-organ involvement, including the heart. For such high-risk induction, an agent capable of preserving vascular tone and myocardial contractility - while imposing no additional insult to already compromised organs - is mandatory. A 2024 meta - analysis of 13 RCTs by Akhtar et al. (11) demonstrated that ciprofol provides hypnosis equivalent to propofol, and lowers the incidence of induction-related hypotension by 18% (RR 0.82, 95% CI 0.68–0.98). In ECD patients with cardiac infiltration, even a transient drop in afterload from propofol could precipitate circulatory collapse; the smaller hypotension risk therefore clearly favors ciprofol. Etomidate was avoided because repeated boluses or continuous infusion suppress adrenal function for >24 h, and our patient’s central diabetes insipidus left the hypothalamic - pituitary - adrenal axis reserve uncertain. Taken together, ciprofol offered the smoothest loss of consciousness with the least added injury to failing organs, making it the rational induction choice in this case. We adopted an intra - operative lung - protective ventilation bundle (tidal volume 7 mL/kg ideal body weight, PEEP 5 cm H2O) throughout the case. These settings are consistent with the 2019 international expert-consensus recommendations for surgical patients (12), which propose initial tidal volume 6–8 mL/kg predicted body weight and PEEP ≥ 5 cm H2O to minimize post-operative pulmonary complications and to keep driving pressure as low as feasible. By maintaining low tidal volume and limiting driving pressure, we attempted to attenuate further ventilator - induced lung injury in a patient whose lungs were already compromised by ECD - related interstitial infiltration.

In short, this patient survived because of early assessment, early empiric antibiotics, early CRRT and early multidisciplinary teamwork, enabling discharge to the hematology ward on post-operative day 10. For any ECD patient undergoing emergency surgery, the strategy must revolve around precise balancing of “low organ reserve - immune paralysis - fluid shifts” superimposed upon an already rare and complex disorder.

## 4 Conclusion

We reported a case in which multidisciplinary perioperative management safely facilitated emergency appendectomy in a patient with Erdheim - Chester disease and multiorgan involvement. Early assessment, empiric antibiotics, CRRT, and multidisciplinary teamwork have been the core strategies of this case, but validation in a larger cohort is still needed.

## Data Availability

The original contributions presented in this study are included in this article/supplementary material, further inquiries can be directed to the corresponding author.
